# Does unrestrained single-chamber plethysmography provide a valid assessment of airway responsiveness in allergic BALB/c mice?

**DOI:** 10.1186/1465-9921-10-61

**Published:** 2009-07-03

**Authors:** Qingling Zhang, Kefang Lai, Jiaxing Xie, Guoqin Chen, Nanshan Zhong

**Affiliations:** 1 State Key Laboratory of Respiratory Disease (Guangzhou Medical University), The First Affiliated Hospital of Guangzhou Medical University, Guangzhou, PR China; 2 Department of Pathology, The First Affiliated Hospital of Guangzhou Medical University, Guangzhou, PR China

## Abstract

**Background:**

Unrestrained plethysmography has been used to monitor bronchoconstriction because of its ease of use and ability to measure airway responsiveness in conscious animals. However, its reliability remains controversial.

**Objective:**

To investigate if unrestrained plethysmography could provide a valid interpretation of airway responsiveness in allergic BALB/c mice.

**Methods:**

Ovalbumin sensitized BALB/c mice were randomized to receive either a single-dose Ovalbumin challenge (OVA-1D group) or a three-dose Ovalbumin challenge (OVA-3D group). The OVA-1D group was further divided into OVA-1D-I (measured invasively, using lung resistance as the index of responsiveness) and OVA-1D-N group (measured non-invasively, using Penh as the index of responsiveness). Similarly the OVA-3D group was divided into OVA-3D-I and OVA-3D-N groups based on the above methods. The control groups were sensitized and challenged with normal saline. Bronchial alveolar lavage fluid was taken and airway histopathology was evaluated for airway inflammation. Nasal responsiveness was tested with histamine challenge.

**Results:**

Compared with controls, a significant increase in airway responsiveness was shown in the OVA-1D-N group (P < 0.05) but not in the OVA-1D-I group. Both OVA-3D-I and OVA-3D-N groups showed higher responsiveness than their controls (P < 0.05). The nasal mucosa was infiltrated by eosinophic cells in all Ovalbumin immunized groups. Sneezing or nasal rubbing in allergic groups appeared more frequent than that in the control groups.

**Conclusion:**

Penh can not be used as a surrogate for airway resistance. The invasive measurement is specific to lower airway. Penh measurement (done as a screening procedure), must be confirmed by a direct invasive measurement specific to lower airway in evaluating lower airway responsiveness.

## Background

Airway hyperresponsiveness (AHR) is a functional abnormality characteristic of bronchial asthma [1]. AHR in asthma is defined as an exaggerated response of the airway (lower airway in particular) to a variety of nonspecific stimuli, resulting in airway obstruction [2,3].

Several measurement techniques which have been used for the investigation of airway responsiveness (AR) in mice in vivo include invasive and non-invasive approaches [4]. Invasive measurements of pulmonary function are performed in tracheotomized, endotracheally intubated rodents or in orotracheally intubated rodents. These involve the determination of airway resistance and dynamic compliance, which are the gold standards in assessing bronchoconstriction. Recently, unrestrained barometric plethysmography in conscious mice or rats represents the extreme of non-invasiveness and has been widely used for measuring airway hyperresponsiveness in murine models of allergic airway inflammation [[5-8], and [9]]. It is attractive because of its ease of use and its ability to obtain data rapidly and non-invasively, especially in conscious animals. However, controversy remains on its validity to the measurement of airway responsiveness [10-17] and so far, there has not been sufficient data supporting Penh as a surrogate for airway resistance [18].

For an insight into the controversy, we measured allergic mice by both non-invasive and invasive methods, and compared constriction data measured by Penh to resistance measurements done invasively.

## Methods

### Animals

One hundred and twenty pathogen-free, female BALB/c mice, 6–7 weeks of age, 18–20 g body weight, were purchased from Animal Experiment Center of Guangzhou University of Chinese Medicine. Upon delivery, the mice were kept in a pathogen-free rodent facility and were provided food and water ad libitum. The animal experiments were approved by Animal Experiment Centre of Guangzhou University of Chinese Medicine.

### Sensitization and Airway Challenge

Test mice were sensitized systemically with ovalbumin (OVA 10 ug/injection, grade V, Sigma, St Louis, MO, USA) adsorbed to 1.3 mg of aluminum hydroxide gel [Al(OH)3, Sigma, USA] by intraperitoneal injections on days 0, 7 and 14. Test mice were challenged by intranasal instillation of OVA either once on day 28; or three times, once daily on each of days 28, 29, and 30. 2 mg OVA was dissolved in 1 ml sterile saline and instilled intranasal into the mice (100 ug/50 ul OVA solution, 2_per mouse) using a sterile pipette. Control mice were sensitized and challenged with diluents.

**Figure 1:**
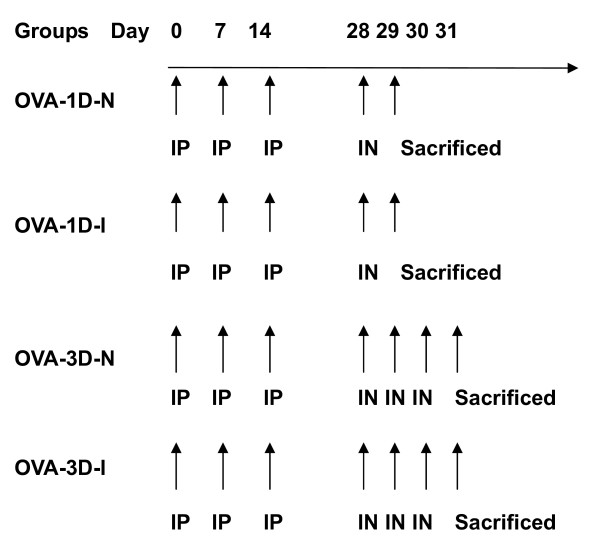
**Protocol for ovalbumin (OVA) intraperitoneal (i.p.) sensitization and subsequent OVA intranasal (i.n.) challenge**. Mice were sensitized by an intraperitoneal injection of 10 μg OVA on days 0, 7 and 14, followed by daily intranasal challenges with 0.2% OVA. OVA-1D-N was challenged on day 28 and airway responsiveness was carried out on day 29 by Penh measurements. OVA-1D-I were challenged on day 28 and airway responsiveness was carried out on day 29 by invasive methods. OVA-3D-N were challenged on days 28, 29, 30 and airway responsiveness was carried out on day 31 by Penh measurements. OVA-3D-I were challenged on days 28, 29, 30 and airway responsiveness was carried out on day 31 by invasive methods.

OVA immunized mice were divided into four groups based on their treatment and measurement of airway responsiveness (see Figure 1).

OVA-1D group (N = 32): Mice were sensitized as described above, and challenged on day 28. On day 29, airway responsiveness was measured. The group was further divided into two sub-groups, namely, the OVA-1D-I group [measured invasively using "RC"system, Buxco, USA] and OVA-1D-N group [measured non-invasively using barometric whole body plethysmography (WBP system, Buxco, USA)].

OVA-3D group (N = 32): Mice were sensitized as described above, and challenged on days 28, 29, and 30. On day 31, airway responsiveness was measured. The group was divided again into two sub-groups: OVA-3D-I group measured invasively, and OVA-3D- N group, measured noninvasively.

Control group((N = 56): Mice were sensitized and challenged with normal saline; using the same volume of solution as used in the OVA-treated mice, applied both i.p. and i.n., respectively.

### Airway Responsiveness Measurement

#### Penh measurements (Non-invasive approach)

Airway responsiveness was assessed using a single-chamber, whole body plethysmograph (WBP system, Buxco, USA) as described by Hamelmann and coworkers [5]. In this system, an unrestrained and spontaneously breathing mouse was placed into the main chamber of the plethysmograph; the plethysmograph was calibrated by injecting 1 ml of air before the measurements. In the plethysmograph, mice were exposed for 1.5 minutes to nebulized normal saline (Aerogen nebulizer head, particle size 4–6 um mass median aerodynamic diameter, licensed by Buxco, USA) and subsequently to increasing concentrations of nebulized MCh (0.39–50 mg/ml; Sigma, USA). When the animal inspires, air is removed from the chamber, and enters the lungs, driving the chamber pressure down (nasal flow) at the same time, however, the lungs expand, increasing the chamber pressure (thoracic flow). The thoracic expansion on inspiration is always greater than the volume of air withdrawn from the chamber, for two reasons: First, thermodynamic effects come into play. The air from the chamber is heated and humidified once it is in the animal. Therefore the increase in chest or thoracic volumes somewhat larger than the air removed from the chamber through the nose. Secondly, there may be compression and rarefaction effects within the lungs due to effort of breathing, and these effects may be more prominent in particular regions of the respiratory cycle. If there is an obstruction, or a constriction in the airways, and the musculature moves the thorax, without a concomitant nasal or head flow response, the difference between the chest and nasal flows increases. The difference between the thoracic expansion and the air removed from the chambers creates the respiratory signal (box flow) that is measured in WBP system.

From the box flow signal, we derived: Inspiratory time (TI); expiratory time (TE); relaxation time (TR), the time for the expiratory area to decline to 36% of the total expiratory area; peak inspiratory flow(PIF) and peak expiratory flow (PEF); tidal volume (VT); minute ventilation (VE); and respiratory rate (RR); Pause (= [TE-TR]/TR); and Penh (=pause PEF/PIF). Penh is considered an empiric parameter that reflects changes in waveform of the measured box pressure signal as consequence of bronchoconstriction. After the end of aerosolization, the Penh values were measured during each 3-min sequence, as well as the mean of each MCh concentration, and presented as the percent changes from corresponding baseline values. The provocative concentrations of the agonist that increased Penh to 200% and 300% of baseline (PCPenh200, PCPenh300) were obtained by linear interpolation of the concentration response curve between the final two doses of the respective provocative agent [19,20]. There were 12 untreated mice tested by Penh measurements for each subgroup.

#### Invasive approach

Using the invasive measurement system ("RC" system, Buxco, USA), pulmonary measurements are performed in tracheotomized, endotracheally intubated (stainless steel cannula, 18 gauge) mice. These techniques had been described before [21]. Briefly; mice were anesthetized with intraperitoneal injections of sodium pentobarbital (70 to 90 mg/kg body weight) with minimal supplementations as required. When an appropriate depth of anesthesia was achieved, as monitored by a loss of the righting and pinch toe reflex, mice were tracheotomized, endotracheally intubated and connected to a ventilator (Model 683, Harvard, USA), then ventilated with a tidal volume of 180–190 ul and a respiratory frequency of 125 times of breath. The animals were then placed supine in a whole body plethysmograph. The endotracheal tube was connected to a manifold with three multiple ports outside the chamber: two ports for connections to the ventilator, and one port to a differential pressure transducer for monitoring of tracheal pressure. Esophageal pressure was monitored via water filled tubing (CNS1010, Buxco, USA), and connected to the other port of the differential pressure transducer. Thus transpulmonary pressure, Ptp, (tracheal – esophageal), was monitored and used in the computations. The esophageal tubing was inserted to the level of the midthorax. The optimal position of the tube was in the lower third of the esophagus where we monitored maximum negative pressures.

Airflow was monitored by a pneumotachograph in the wall of the plethysmograph. The pressure within the plethysmograph monitored the flow due to the animal's thoracic movements. Lung resistance was determined from the ratio of Ptp to tidal flow over an entire breath cycle. The signals of flow and transpulmonary pressure were recorded on a computer. Respiratory volume was obtained by digital integration of the flow signal so that RL (lung resistance) was calculated from the transpulmonary pressure and flow at isovolumetric points. After the end of aerosolization, the RL values were measured during each 3-min sequence as the mean for each MCh concentration, and presented as the percent changes from corresponding baseline values. Before each experiment, calibrations of flow and pressure were performed with a volume of 1 ml of air and pressures of 0 and 20 cmH_2_O, respectively.

### Lung Pathological Analyses

#### Bronchoalveolar Lavage Analysis

After AR measurements, animals were euthanized by injection with a lethal dose of a pentobarbital-based euthanasia solution. Blood was collected by cutting the renal artery. Their tracheas were cannulated, and their chests were opened. Bronchoalveolar lavage (BAL) cells were obtained by inserting a catheter into the trachea and lavaging the lung three times with 0.8 ml of phosphate-buffered saline (PBS). Approximately, 2.0 ml BAL fluids was consistently recovered with gentle handling. The retrieved lavage aliquots were pooled and centrifuged at 4 degrees Celsius, 1500 rpm for 10 min, from which the cell pellet was resuspended in PBS and counted using a hemocytometer. Slide smears were treated with hematoxylin-eosin stain (Sigma, USA) for differential cell counts with at least 300 leukocytes in each sample. Stained slides were read randomly and in a blinded manner. The cell types were judged according to standard hemocytologic procedures as neutrophils, eosinophils, lymphocytes, or macrophages. The data were obtained from seven mice per group after AR measurement.

#### Bronchial histopathology

After blood collection, some animals had their lungs instilled via the trachea with 10% buffered formalin, removed, and fixed in the same solution. Animals used for histopathologic analysis were not subjected to BAL. After paraffin embedding, sectioned at 4 um, and stained with hematoxylin and eosinphloxin (H&E), histopathological assessment (light microscopy) was performed blind on randomized sections. Inflammatory changes were graded using a semiquantitative scale of 0–5 for perivascular eosinophilia, bronchiolar eosinophilia, epithelial damage and oedema as previously described [22]: scale of 0 (none), 1 (minimal), 2 (mild), 3 (moderate), and 4 (severe). Individual lesion scores were summed from each animal to create an overall histopathology score for each animal. A pathologist who was blinded to the exposure conditions evaluated all slides. The data were obtained from four to five mice per group after AR measurement.

### Analysis of upper airway

#### Nasal challenge with histamine

In another test, OVA-1D and OVA 3D mice (and their controls) which followed the study design as described before, were challenged with histamine (n = 8 for each group). Because mice are preferential nose breathers, small droplets of solution were placed on the external naris of awake mice to be drawn into the nasal passages during inhalation. As described by Klemens J [23], the nasal challenge with histamine consisted of intranasal application of 50 μl of various concentrations of histamine (Sigma, USA) applied gradually over 2 minutes. The challenge involved 3 exposures to histamine (0.3 mM, 3.0 mM, and 30 mM). After each exposure, allergen-induced nasal symptoms were evaluated by counting the number of sneezes and nasal itching motions (nasal rubbing) that occurred within a 10-min interval by the same investigator who was blinded to the treatment groups.

#### Infiltration of Eosinophils in Nasal Mucosa and nasopharynx Mucosa

**Figure 2:**
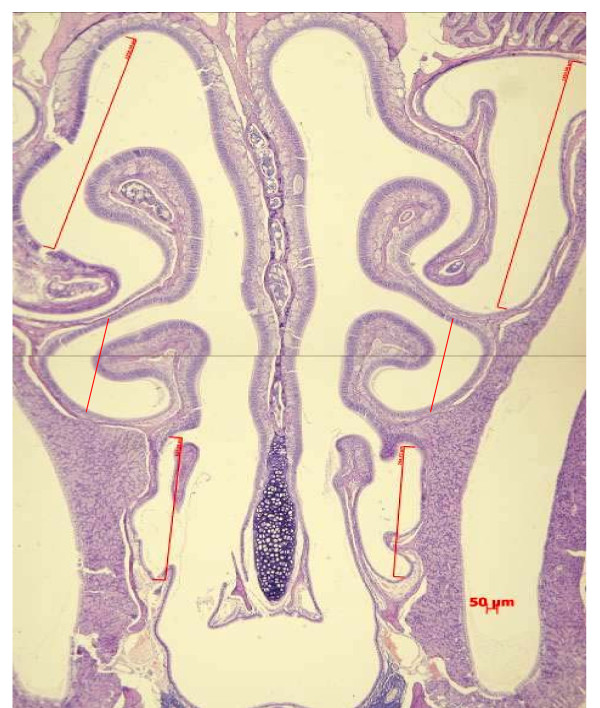
**Light microscopic images of the murine nose (50 fold magnification) of a coronal section through the sinonasal skeleton, showing the murine nasal anatomy**. Eosinophils were counting in a defined region of the nasal mucosa (along red line).

After AR measurements by Penh measurements, the mice were skinned, fixed for 48 h in buffered formalin (10%) at 20 degrees Celsius and decalcified during 2 weeks using a 14% ethylenediamineteraacetic acid (Sigma, USA) solution. Coronal sections of the skulls in the middle and the third between nose-tip and orbit were made and stored in formalin until further processing. After dehydration and embedding in paraffin, a thickness of 4 μm, the specimens were then deparaffinized and stained with hematoxylin-eosin. Representative nasal sections were scored by counting eosinophils in (sub)-epithelial layers of both lateral nasal walls by using an eyepiece reticule. The number of eosinophils was quantified per unit (1 mm^2^) of lateral nasal walls length (between the lower edge of the upper turbinate and the upper edge of the middle turbinate; the lower edge of the middle turbinate and the upper edge of the lower turbinate; the lower edge of the lower turbinate and nose-tip) (Figure 2). The data were obtained from nine to ten mice per group after AR measurement by non-invasive approach.

### Statistical Analysis

For all cell counts, stained slides were read randomly and in a blinded manner. All statistical analyses were performed using SPSS 12.0 Version package (SPSS Inc., Chicago, IL). The percentage of BAL cells, inflammatory lesion scores of lung and the infiltration numbers of eosinophils in nasal were expressed as median and interquartile range (IQR). Normal-distributed data were compared using analysis of variance (ANOVA) or unpaired t test, whereas the non-parametric Kruskal-Wallis test was used otherwise. If significant differences have been found, Bonferroni test was used as a multiple comparative test to evaluate the differences in nasal and lower airway hyper-responsiveness. All hypothesis testing was two-sided and P < 0.05 was defined as significant.

## Results

### Airway Responsiveness

There were 12 untreated mice tested for lung mechanics in each group. One mouse in each of OVA-1D-I group and Control group died during cannulating trachea. One mouse in OVA-3D-I group died of anesthesia. These mice were not included in the analysis. 93 mice completed the test satisfactorily.

**Figure 3:**
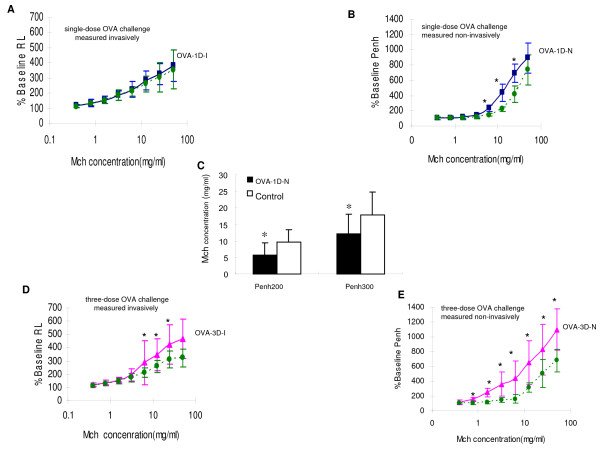
**24 hours after final exposure, mice were assessed for airway responsiveness to Mch challenge**. (A), (B) and (C) are OVA sensitized with single-dose OVA challenge, measured invasively (A, OVA-1D-I, squares on solid line) or by Penh measurements (non-invasively) (B, OVA-1D-N, squares on solid line). (D) and (E) are OVA sensitized with three-dose OVA challenge, measured invasively (D, OVA-3D-I, triangles on solid line) or by Penh measurements (E, OVA-3D-N, triangles on solid line). *P < 0.05 compared with controls (circles on dotted line).

24 hours after final exposure, mice were assessed for AR by Mch challenge. The OVA-1D -I group, measured invasively for resistance, did not have an increase in AR in comparison to control mice (see Figure 3A). In contrast, the OVA-1D- N group which had the same allergen sensitization and challenge protocol as OVA-1D-I group showed a significant increase in AR at MCh concentrations of 6.25 – 25 mg/ml as measured by Penh measurements (see Figure 3B). In addition, Penh 200 and Penh 300 decreased significantly in OVA-1D- N group when compared with the control group (see Figure 3C). Both OVA-3D-N and OVA-3D-I groups presented with airway hyperresponsiveness (see Figures 3D, E). Compared with the control group, airway hyperresponsiveness in OVA-3D-N group was shown at MCh concentrations of 0.78–50 mg/ml but at MCh concentrations of 12.5 – 50 mg/ml in OVA-3D-I group.

### Lung Pathological Analyses

#### Bronchoalveolar Lavage Analysis

There was no difference in the percentage of BAL cells between the controls of all four test groups. The control data for the OVA-1D-I group was chosen for comparison to all test groups. As indicated in Table 1, the percentage of BAL neutrophils and eosinophils increased and the percentage of BAL macrophages significantly decreased in all OVA immunized mice when compared with the control group. The percentage of eosinophils in BALF of OVA-3D-I group was significantly higher than that of OVA-1D-I group (P < 0.05). There were no differences in the percentage of BAL lymphocytes, neutrophils, macrophages and eosinophils between OVA-1D-I and OVA-1D-N group or between OVA-3D-I and OVA-3D-N group (see Table 1).

**Table 1:** Median (IOR) differential cell counts (%) in BALF

	Macrophages (%)	Lymphocytes (%)	Neutrophils (%)	Eosinophils (%)	Total Cells (*10^5^/ml)
OVA-1D-I	42.79(37.61)	12.50(17.85)	21.63(36.20)	23.20(18.42)	2.90(1.10)
OVA-1D-N	37.95(22.99)	6.50(2.10)	27.57(13.10)	23.20(15.17)	2.70(1.00)
OVA-3D-I	32.27(33.68)	7.17(11.89)	11.16(9.10)	40.36(18.54)^†^	7.30(2.70)^††^
OVA-3D-N	30.41(3.58)	8.00(16.76)	8.57(12.20)	48.71(11.70)	8.80(3.50)
Control(I)	89.13(12.54)*	7.81(17.46)	0.93(3.20)*	0.00(0.00)*	1.10(0.50)*

All mice were sensitized with OVA (10 ug, i.p) on days 0, 7 and 14. Mice in OVA-1D group were single-dose challenged on day 28. On day 29, responsiveness of mice was carried out with invasive measurements (OVA-1D-I group) or non-invasive measurements (OVA-1D-N group). Mice in OVA-3D group were three-dose challenged on day 28, 29, 30. On day 31, responsiveness of mice was carried out by invasive (OVA-3D-I group) or Penh measurements (OVA-3D-N group). The mice in Control (I) group were sensitized and challenged with normal saline.

*P ≤ 0.01 for Control group versus OVA immunized group, Kruskal-Wallis test.

^†^*P *< 0.05 for OVA-3D-I group versus OVA-1D-I group, Kruskal-Wallis test.

^††^*P *≤ 0.001 for OVA-3D-I group versus OVA-1D group (single-dose OVA challenge), Kruskal-Wallis test.

#### Bronchial histopathology

**Figure 4:**
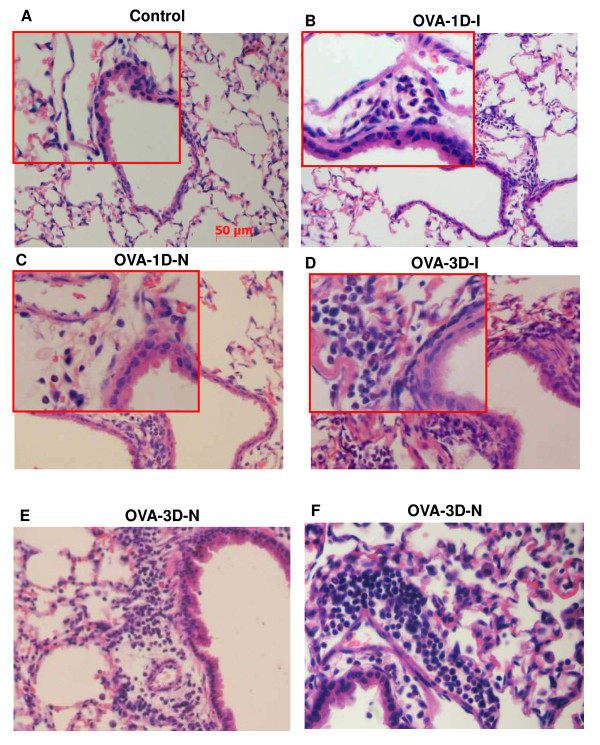
**Representative hematoxylin and eosin-stained lung sections collected after assessment of pulmonary mechanics from mice**. (B) and (C) is OVA sensitized with single-dose OVA challenge, measured invasively (B) or by Penh measurements (C). (D), (E) and (F) are OVA sensitized with three-dose OVA challenge, measured invasively (D) or by Penh measurements (E, F).

There was no difference in inflammatory lesion scores between the controls of all four test groups. The control data for the OVA-1D-I group was chosen for comparison to all test groups. There were no differences in inflammatory lesion scores between the OVA-1D-I and the OVA-1D-N group (see Table 2, Figure 4B and 4D) or between the OVA-3D-I and the OVA-3D-N groups. Mice in the OVA-3D group had higher inflammatory lesion scores than those in the OVA-1D group (see Table 2, Figure 4B–F). Inflammation in OVA-1D mice consisted of minimal-to-mild inflammation in peribronchiolar and perivascular interstitial infiltrates of eosinophils, neutrophils and macrophages mixed with occasional plasma cells and rare lymphocytes (see Figure 4B and 4C) while OVA-3D mice showed moderate-to-severe inflammatory responses in the peribronchovascular connective tissue sheaths surrounding arteries and airways (see Figure 4D, E and 4F). Moreover, in OVA-3D mice, macrophages and eosinophils occasionally widened alveolar septa slightly in the parenchyma (see Figure 4F) and a few eosinophils along with a few macrophages were present in alveolar septa (either interstitial or within the capillary bed).

**Table 2:** Inflammatory lesion scores in OVA immunized group and control group

Different group	Perivascular eosinophilia	Peribronchiolar eosinophilia	Edema	Epithelial damage
OVA-1D-I	3(1–3)	1(1–2)	1(1–2)	1(1–3)
OVA-1D-N	3(2–3)	1(1–2)	1(1–2)	1(1–3)
OVA-3D-I	4(3–5)^†^	2.5(2–3)^†^	1(1–2)	2(1–3)
OVA-3D-N	3.5(3–5)^†^	2(2–3)^†^	1(1–2)	2(1–3)
Control(I)	0(0–0)*	0(0–0)*	0(0–0)*	0(0–1)*

All mice were sensitized with OVA (10 ug, i.p) on days 0, 7 and 14. Mice in OVA-1D group were single-dose challenged on day 28. On day 29, responsiveness of mice was carried out with invasive measurements (OVA-1D-I group) or non-invasive measurements (OVA-1D-N group). Mice in OVA-3D group were three-dose challenged on day 28, 29, 30. On day 31, responsiveness of mice was carried out by invasive (OVA-3D-I group) or Penh measurements (OVA-3D-N group). The mice in Control (I) group were sensitized and challenged with normal saline.

* *P *< 0.05 for Control group versus OVA immunized group, Kruskal-Wallis test.

^†^*P *< 0.01 for OVA-3D group versus OVA-1D group, Kruskal-Wallis test.

### Analysis of upper airway

#### Nasal challenge with histamine

**Figure 5:**
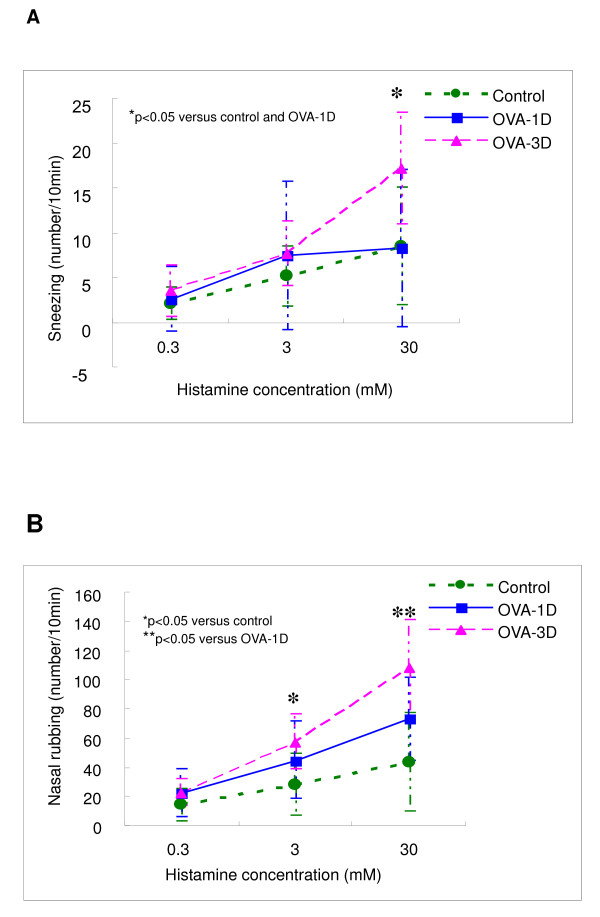
**Mean ± SD of sneezes(A)and nose rubs (B) after various concentrations of intranasal histamine in mice**. The mice were sensitized with OVA and challenged with single-dose OVA challenge (OVA-1D, blue color line) or three-dose OVA challenge (OVA-3D, red color line). *P < 0.05 compared with control (green color line). **P < 0.05 compared with OVA-1D.

As shown in Figure 5, the number of sneezes in OVA-3D mice was significantly higher than that in OVA-1D mice and control mice for 30 mM-histamine. In OVA-1D mice, the number of nose rubs was significantly higher than control mice for 30 mM-histamine. In addition, the number of nose rubs in OVA-3D mice was significantly higher than control mice for 3 and 30 mM-histamine and higher than OVA-1D mice just at 30 mM-histamine.

#### Infiltration of Eosinophils in Nasal and Pharyngeal Portion Mucosa

**Figure 6:**
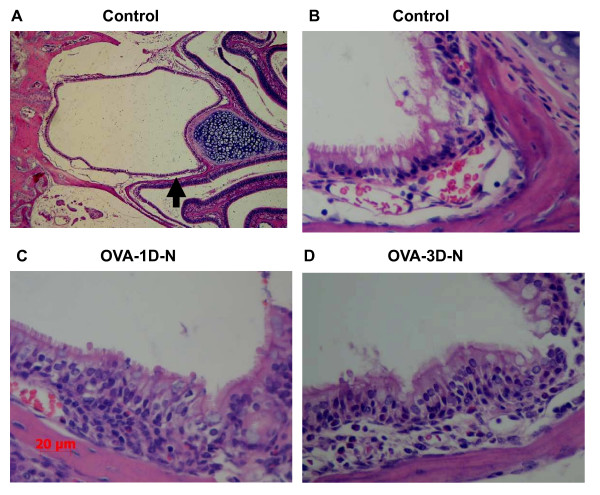
**Representative hematoxylin and eosin-stained pharyngeal portion mucosa collected after airway responsiveness measured by Penh measurements**. (A) Light Microscopic image (50 fold magnification) of Control mice. (B) Magnification of (A), 400 fold magnification. (C) OVA sensitized with single-dose OVA challenge, airway responsiveness was carried out by Penh measurements. (D) OVA sensitized with three-dose OVA challenge, airway responsiveness was carried out by Penh measurements.

Both in OVA-1D group and OVA-3D group, there was obvious inflammation in upper airway [see figure 6].

On day 29 after assessment of pulmonary mechanics, upper airway histology showed mild inflammatory responses in pharyngeal mucosa of OVA-1D group mice, with mild infiltration of few eosinophils and neutrophils. On day 31 after assessment of pulmonary mechanics, mild to moderate inflammation could be seen in OVA-3D group mice with mild to moderate infiltration of eosinophils, neutrophils mixed with occasional macrophages.

**Figure 7:**
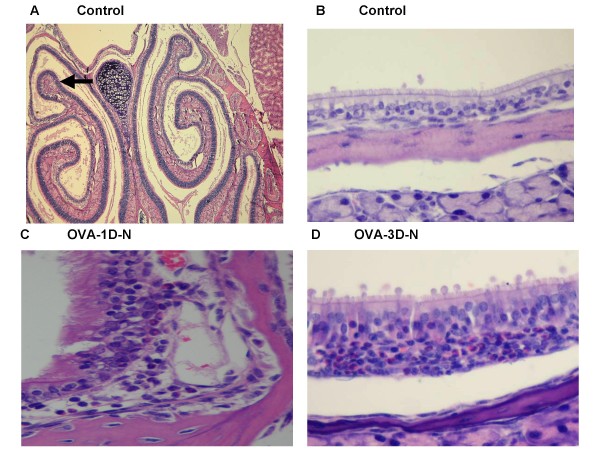
**Representative hematoxylin and eosin-stained nasal mucosa collected after airway responsiveness measured by Penh measurements.** (A) Light Microscopic image (50 fold magnification) of Control mice. (B) Magnification of (A), 400 fold magnification. (C) OVA sensitized with single-dose OVA challenge, airway responsiveness was carried out by Penh measurements. (D) OVA sensitized with three-dose OVA challenge, airway responsiveness was carried out by Penh measurements.

The nasal inflammatory infiltrate in OVA immunized mice was present mainly in the subepithelial layer along the edge of the upper turbinate and consisted primarily of infiltrating eosinophils and mononuclear cells (see Figure 7). The median number of eosinophils in the nasal sub-epithelium in both OVA immunized groups were higher than in the control group (0.00/um, range 0–5.03) (P > 0.05), but there were no differences between OVA-1D-N group (median 10.55/um, range 4.16–33.62) and OVA-3D-N group (median 6.69/um, range 1.11–48.91).

## Discussion

Traditional invasive pulmonary function tests have been shown to be sensitive in detecting bronchoconstriction in mice. This method appears precise and specific, because the nasal exposure is excluded, thus focusing on the inhalation exposure to the lungs. However, the need for anesthesia and invasive procedures such as tracheotomy or orotracheal intubation, and mechanical ventilation makes this approach a study of animals under conditions far from natural [11,13].

On the other hand, the highly reproducible Penh measurements seem to be suitable for repeated pulmonary measurements, e.g. in long-term follow-up studies, or in asthma models with assessment of early airway response and late airway hyperresponsiveness in the same animal. However, its validity remains controversial. Albertine et al [14] and Petak et al [15] have shown that there is an inconsistent relationship between Penh and invasive measurements, especially in C57BL/6 mice. Enhorning et al [16], Lundblad et al [17] and Mitzner et al [24] have shown that the relationship between the chamber pressure, from which Penh is calculated, and the airway resistance is limited. Hamelmann et al [5], demonstrated that the responsiveness of allergen-sensitized mice to methacholine, as measured with the WBP system, paralleled the responsiveness of airway resistance, as measured invasively. He concluded that the non-invasive barometric plethysmography provided a new opportunity to evaluate the mechanism and kinetics underlying the development and the maintenance of airway responsiveness. However, in his study, Penh and RL were not obtained on the same day of the protocol (Penh was measured on Day 31 of the protocol and RL was obtained on day 32). In our experience, eosinophils cell counts in bronchial alveolar lavage fluid and airway histopathology (data not shown) presented different airway inflammation on different days of the protocol.

Because there are not currently sufficient data as to conclude whether Penh could be used to detect and measure airway responsiveness, we used both Penh and invasive measurements to investigate AR in BALB/c mice which had undergone the same sensitization and challenge protocol. As shown in our results, the OVA-1D group had mild inflammation while the OVA-3D group had severe inflammation in the lung. In contrast to Penh measurements, the invasive measurement showed no increase in AR compared to control mice for the mild inflammation group. Furthermore, compared with the control group, airway hyperresponsiveness measured by Penh measurements in severe inflammation group was found in lower MCh concentrations than that which measured by invasive measurements.

Some experts argue that anesthesia or the trauma of intubations may affect, to some extent, the airway responsiveness. But it should be noted that in this study, the comparison was only made between the allergic mice and the control mice in the same condition.

At the time of Penh measurements, the number of sneezes or nasal itching in most allergic mice in response to Mch was significantly higher in comparison to control mice. So we hypothesized that Penh value in unrestrained plethysmography in conscious mice might include the contribution of upper airway resistance and it could reflect airflow limitation in the upper airway, including the nasal cavity, larynx and pharynx, etc. As we know, in humans, nasal resistance contributes about 50% of the respiratory resistance [5]. When AR is measured in humans, Mch or histamine is usually inhaled via mouth and nose clips are used during the testing. However, it is impossible for mice to adapt to such a protocol.

In addition, we had further analyzed the characteristics of the upper airway of the experiment mice, including the histology and nasal challenge with histamine. The sneezing or nasal itching in airway allergic groups was significantly more frequent than that in control groups. The histological study clearly showed infiltration of inflammatory cells in the nasal mucosa of allergic mice. The present findings suggested that airflow limitation in the upper airway, including the nasal cavity, might affect the value of Penh, and include an uncertainty in the exact magnitude of bronchoconstriction. Our results demonstrate that the increased upper airway resistance is the major factor influencing Penh, therefore, the changes in Penh may not be a reliable indicator of change of lower airway responsiveness, at least in mild Ovalbumin sensitized BALB/c mice.

A recent report shows that Penh can be influenced by increases in upper airway resistance, while the lower airway was unaffected in their model. Nakaya et al [25] has described an application of the Penh system to study nasal hypersensitivity, suggesting that the non-invasive system could be very useful in the study of nasal hypersensitivity. Furthermore, Taw Tsumuro et al[26] evaluated nasal congestion in rats using whole body plethysmography, and noted that Penh increased significantly following the intranasal application of histamine in Toluene-2, 4-diisocyanate (TDI) sensitized rats, indicating the changes in Penh, induced by TDI challenge reflected upper airway congestion in their model. Anurag et al [27], Using Double-chamber plethysmography, showed that nasal resistance change comprises one-half or more of the total resistance change during methacholine challenge.

The other possibility we need to point out is that mice are preferential but not obligate nasal breathers [27]. After nasal occlusion, most mice switch to an oral mode of breathing with no apparent discomfort. Histamine may lead to a change in the pattern of breathing and then it can also make Penh well changed.

This study showed that invasive and Penh measurements might lead to the different results for airway responsiveness in the same mildly allergic mice group. One of the explanations may be that the airway inflammation of the mild allergic mouse was comparatively mild, which lead to modest bronchoconstriction in the lower airway. It had been shown that not all airway inflammation leads to airway hyperresponsiveness. For example, nonasthmatic eosinophilic bronchitis (NAEB) is a newly recognized cause of chronic cough in human [28]. It is characterized by the presence of eosinophilic airway inflammation, similar to that seen in asthma. However, in contrast to asthma, nonasthmatic eosinophilic bronchitis is not associated with variable airflow limitation or airway hyperresponsiveness [29]. Another explanation may be that inhalation exposure includes nasal and gastro-intestinal uptake by Penh measurements. Penh may pick up all sources of resistance, upper airway as well as lower airway, and it might not necessarily represent a change in the lower respiratory tract. The increased airway responsiveness in Penh measurements may be related to obstruction of upper airway but not of lower airway in some models. In contrast to mildly allergic models, the change of Penh in moderately or severely allergic mice may be derived from both upper and lower airway resistance.

Based on our study, at least in mild ovalbumin-sensitized BALB/c mice, Penh cannot be used as a surrogate for airway resistance when sensitivity to cholinergic stimulation is studied. It is likely that Penh contains upper airway resistance components as well as lower airway resistance components. It is not clear how much is upper airway resistance and how much is lower airway resistance. Such an effect is bypassed by the tracheotomy or orotracheal intubations in the invasive measurement. Therefore, in evaluating lower airway responsivity, a Penh measurement (done as a screening procedure), must be confirmed by a direct invasive measurement specific to lower airway.

## Conclusion

In mildly allergic mice, the increased airway resistance as shown with non-invasive measurement may be due to upper airway resistance. In moderately or severely allergic mice, the increased airway resistance may be derived from both upper and lower airway. The invasive measurement is specific in measuring lower airway resistance.

## Abbreviations

AHR: Airway hyperresponsiveness; AR: Airway responsiveness; RL: Lung resistance; BAL: Bronchoalveolar lavage; H&E: Hematoxylin and eosinphloxin.

## Competing interests

None of the authors has a commercial or other association that might pose a conflict of interest.

## Authors' contributions

QLZ and KFL made the same contributions for this paper, they wrote the manuscript, carried out the establishment of allergic animal model and study planning, performed laboratory work and statistical analyses. JXX carried out the animal studies and assisted with airway responsiveness measurement. GQC carried out the evaluation of airway inflammation. NSZ provides overall leadership to the design of the experiments, data analysis, and preparation of the manuscript. All authors participated in manuscript design and revisions and approved the final manuscript.
